# The Ability of ChatGPT in Paraphrasing Texts and Reducing Plagiarism: A Descriptive Analysis

**DOI:** 10.2196/53308

**Published:** 2024-07-08

**Authors:** Soheil Hassanipour, Sandeep Nayak, Ali Bozorgi, Mohammad-Hossein Keivanlou, Tirth Dave, Abdulhadi Alotaibi, Farahnaz Joukar, Parinaz Mellatdoust, Arash Bakhshi, Dona Kuriyakose, Lakshmi D Polisetty, Mallika Chimpiri, Ehsan Amini-Salehi

**Affiliations:** 1 Gastrointestinal and Liver Diseases Research Center, Guilan University of Medical Sciences, Rasht, Iran; 2 Department of Internal Medicine, Yale New Haven Health Bridgeport Hospital, Bridgeport, NY, United States; 3 Tehran Heart Center, Tehran University of Medical Sciences, Tehran, Iran; 4 Department of Internal Medicine, Bukovinian State Medical University, Chernivtsi, Ukraine; 5 Department of Medicine, Vision Colleges, Riyadh, Saudi Arabia; 6 Dipartimento di Elettronica Informazione Bioingegneria, Politecnico di Milano, Milan, Italy; 7 Department of Internal Medicine, St. Joseph's Mission Hospital, Anchal, Kollam District Kerala, India; 8 UCLA Neuroscience, Los Angeles, CA, United States

**Keywords:** ChatGPT, paraphrasing, text generation, prompts, academic journals, plagiarize, plagiarism, paraphrase, wording, LLM, LLMs, language model, language models, prompt, generative, artificial intelligence, NLP, natural language processing, rephrase, plagiarizing, honesty, integrity, texts, text, textual, generation, large language model, large language models

## Abstract

**Background:**

The introduction of ChatGPT by OpenAI has garnered significant attention. Among its capabilities, paraphrasing stands out.

**Objective:**

This study aims to investigate the satisfactory levels of plagiarism in the paraphrased text produced by this chatbot.

**Methods:**

Three texts of varying lengths were presented to ChatGPT. ChatGPT was then instructed to paraphrase the provided texts using five different prompts. In the subsequent stage of the study, the texts were divided into separate paragraphs, and ChatGPT was requested to paraphrase each paragraph individually. Lastly, in the third stage, ChatGPT was asked to paraphrase the texts it had previously generated.

**Results:**

The average plagiarism rate in the texts generated by ChatGPT was 45% (SD 10%). ChatGPT exhibited a substantial reduction in plagiarism for the provided texts (mean difference −0.51, 95% CI −0.54 to −0.48; *P*<.001). Furthermore, when comparing the second attempt with the initial attempt, a significant decrease in the plagiarism rate was observed (mean difference −0.06, 95% CI −0.08 to −0.03; *P*<.001). The number of paragraphs in the texts demonstrated a noteworthy association with the percentage of plagiarism, with texts consisting of a single paragraph exhibiting the lowest plagiarism rate (*P*<.001).

**Conclusion:**

Although ChatGPT demonstrates a notable reduction of plagiarism within texts, the existing levels of plagiarism remain relatively high. This underscores a crucial caution for researchers when incorporating this chatbot into their work.

## Introduction

Plagiarism, the act of presenting someone else’s work or ideas as one’s own, stands as a prevalent and recurrent form of misconduct in the field of research and publication [1]. The diverse manifestations of plagiarism can often create confusion due to the various terminologies associated with it. Verbatim plagiarism, mosaic plagiarism, loose plagiarism, duplicate publication, augmented publication, salami-sliced publication, image plagiarism, accidental plagiarism, and self-plagiarism are among the prominent types that have been identified [2-6].

To mitigate the occurrence of such misconduct, researchers often use online plagiarism checkers, which scan existing literature on the internet and provide reports on unintentional plagiarism. Additionally, numerous journals have integrated plagiarism checkers as part of their submission process, wherein every manuscript undergoes scrutiny to identify similarity rates [7]. These measures not only act as deterrents but also aid in upholding the standards of academic integrity and ensuring originality in scholarly publications.

In recent times, artificial intelligence (AI) has gained significant popularity across a wide range of individuals, including researchers and professionals. Among the various applications of AI, chatbots have emerged as a notable development, using AI and natural language processing techniques to generate humanlike responses to user queries [8].

One prominent example of chatbots is ChatGPT, which uses advanced models such as GPT-3.5 and GPT-4. ChatGPT has garnered substantial attention and widespread adoption, amassing over one million users across diverse fields in its first week of launch [9,10]. This surge in popularity reflects the growing recognition and use of AI-powered chatbots in various domains.

ChatGPT offers a multitude of applications and advantages. First, it excels in generating formally structured text, ensuring coherence and organization in its responses. Second, ChatGPT exhibits an extensive and eloquent vocabulary, enhancing the quality and fluency of its generated content. Additionally, it can be used as a rapid search engine, swiftly retrieving relevant information. Furthermore, it possesses the ability to search and analyze available literature, aiding researchers and professionals in their work. In the field of medical education, ChatGPT proves valuable by providing educational resources and facilitating interactive learning experiences. Moreover, it can serve as a conversational agent, engaging in meaningful and interactive conversations with users [10].

Importantly, the text produced by ChatGPT may sometimes bypass conventional plagiarism checks due to its unique generation process, which is a rising ethical concern [10]. Earlier, many researchers were reporting ChatGPT as co-authors in papers but the majority of journals promptly updated their policies to forbid this practice as ChatGPT cannot be held accountable for the generated content [11]. Moreover, in several instances, ChatGPT hallucinates and produces inaccurate and incorrect information, which can be dangerous in academic publishing [12].

Due to the increasing popularity of ChatGPT in medical research, several studies are needed to identify its pros and cons, especially in the field of medical education. In this study, we aim to assess ChatGPT’s real ability to paraphrase and reduce plagiarism by imputing different texts and prompts, and assessing the plagiarism rate of the rephrased texts.

## Methods

### Selection of Texts

To assess the plagiarism rates and the rephrasing capabilities of ChatGPT (version 3.5), three texts were selected for the study. These texts varied in length to provide a comprehensive evaluation of the model’s performance. Text one consisted of 319 words, text two comprised 613 words, and text three encompassed 1148 words. The texts used in this study were selected from one of our previously published medical papers in a medical journal [13].

### Instructions Given to ChatGPT

For each selected text, five distinct prompts were given to ChatGPT to rephrase the texts. These instructions were designed to test different aspects of rephrasing and reducing plagiarism. The prompts are shown in Table 1.

**Table 1.** Prompts provided to ChatGPT.

Number	Prompts
Prompt 1	“Paraphrase the text”
Prompt 2	“Rephrase the text”
Prompt 3	“Reduce the plagiarism of the text”
Prompt 4	“Rephrase it in a way that conveys the same meaning using different words and sentence structure”
Prompt 5	“Reword this text using different language”

### Subdivision of Texts

To further evaluate the effectiveness of ChatGPT in rephrasing and reducing plagiarism, the original texts were subdivided into multiple paragraphs. Specifically, texts one, two, and three were provided to ChatGPT in 1 and 3 paragraphs; 1, 3, and 5 paragraphs; and 1, 3, 5, and 7 paragraphs, respectively. All the texts with different paragraph numbers were subjected to the same five rephrasing orders. This approach allowed for a comparison of the paraphrased texts with different paragraph sections within the same content.

### Second Try of Paraphrasing

To assess the influence of multiple rephrasing iterations, the texts generated by ChatGPT were once again incorporated into the system in the same sequence as before. Subsequently, the plagiarism rates of the texts were analyzed using the iThenticate platform, a tool commonly used for such evaluations in academic settings [14]. This process enabled the measurement and comparison of potential similarities between the original texts and their rephrased counterparts, shedding light on the extent of originality achieved through the rephrasing iterations.

### Data Analysis

The data analysis for this study was conducted using SPSS version 19 (IBM Corp). The data distribution was assessed using the Shapiro-Wilk test. To compare the plagiarism rates of the texts, paired *t* test analysis was used. This statistical test allowed us to examine whether there were significant differences in plagiarism rates between the original texts and the paraphrased texts generated by ChatGPT. Additionally, to assess the potential impact of different prompts on plagiarism rates, 1-way ANOVA was used. This analysis aimed to determine if there were statistically significant differences in plagiarism rates across the various prompts given to ChatGPT. A *P* value <.05 was adopted to determine statistical significance. The acceptable level of plagiarism was set at 25%, a standard embraced by scientific journals. Any plagiarism rate surpassing this threshold was considered unsatisfactory [14-18].

### Ethical Considerations

This study does not require ethical approval as it does not involve human participants, patient data, or any form of personal data collection.

## Results

### Overview

A total of 90 texts were provided by ChatGPT. General information on plagiarism rates is provided in Table 2. The mean plagiarism rate of texts was 0.45 (SD 0.10). The mean plagiarism rates for the first try and second try were 0.48 (SD 0.09) and 0.42 (SD 0.09), respectively.

**Table 2.** Mean plagiarism rates of the texts provided by ChatGPT.

Variable		Text, n	Plagiarism rates checked by iThenticate, mean (SD)
Total		90	0.45 (0.10)
**ChatGPT tries**			
	First try	45	0.48 (0.09)
	Second try	45	0.42 (0.09)
**Texts on the first try**			
	Text 1	10	0.48 (0.16)
	Text 2	15	0.47 (0.05)
	Text 3	20	0.49 (0.07)
**Texts on the second try**			
	Text 1	10	0.46 (0.13)
	Text 2	15	0.40 (0.05)
	Text 3	20	0.42 (0.10)
**Paragraphs**			
	One paragraph	30	0.40 (0.12)
	Three paragraphs	30	0.50 (0.07)
	Five paragraphs	20	0.44 (0.05)
	Seven paragraphs	10	0.48 (0.04)
**Orders given to ChatGPT**			
	Please paraphrase the text	18	0.45 (0.10)
	Please rephrase the text	18	0.48 (0.06)
	Please reduce the plagiarism of the text	18	0.44 (0.10)
	Please rephrase it in a way that conveys the same meaning using different words and sentence structure	18	0.41 (0.12)
	Please reword this text using different language	18	0.48 (0.08)

### The Potency of ChatGPT in Reducing Plagiarism

Based on the results of our study, ChatGPT demonstrated an ability to significantly reduce plagiarism in texts right from the first attempt (mean difference −0.51, 95% CI −0.54 to −0.48; *P*<.001). Moreover, our research revealed that even further improvements were achieved with the second attempt, as it yielded a significantly lower plagiarism rate compared to the initial try (mean difference −0.06, 95% CI −0.08 to −0.03; *P*<.001).

The results also showed a relation between the number of paragraphs within a text and the plagiarism rate. Our findings indicated that texts comprising a single paragraph exhibited the lowest plagiarism rates, and this relationship was statistically significant (*P*<.001). However, when analyzing the five different prompts of the texts, we found no significant difference in terms of their plagiarism rates (*P*=.19).

Furthermore, our study did not identify any statistically significant distinctions among the plagiarism rates of text one, text two, and text three (*P*=.56), suggesting that ChatGPT’s effectiveness remained consistent across these particular texts.

### Correlation Between Text Lengths and Plagiarism Rates

**Figure 1.**
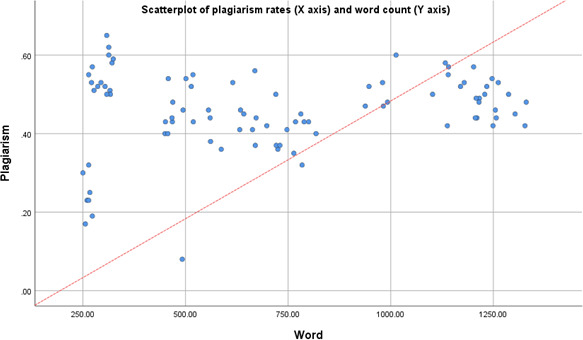
The correlation between the word count of the texts and their corresponding plagiarism.

We assessed the correlation between the word count of the texts provided by ChatGPT and their plagiarism rates. Although longer texts appeared to have higher plagiarism rates, the correlation was not significant (*r*=0.2; *P*=.06; Figure 1).

## Discussion

### Principal Findings

The findings of our study shed light on the levels of plagiarism in the paraphrased text generated by ChatGPT, an advanced chatbot developed by OpenAI. The results indicate that while ChatGPT has the capability to paraphrase the text, there are notable concerns regarding the satisfactory levels of plagiarism in the generated output.

The average plagiarism rate observed in the texts generated by ChatGPT was found to be 45%. This suggests that nearly half of the content produced by the chatbot is similar to the original source material, raising concerns about the authenticity and originality of the paraphrased text. These findings highlight the need for caution when relying on ChatGPT for generating plagiarism-free content.

Interestingly, our study revealed that ChatGPT exhibited a substantial reduction in text plagiarism when provided with explicit instructions to paraphrase or reduce plagiarism. This indicates that the chatbot is responsive to such prompts and can generate content with reduced plagiarism when specifically instructed to do so. However, it is important to note that even with explicit instructions, the plagiarism rate remained relatively high, emphasizing the limitations of the current system.

We also observed a significant decrease in the plagiarism rate between the initial and second attempts of paraphrasing. This suggests that ChatGPT has the ability to learn and improve its paraphrasing capabilities over multiple iterations. However, the reduction in plagiarism was modest, indicating that further refinements are necessary to achieve satisfactory levels of originality in the generated output.

An interesting finding from our study was the association between the number of paragraphs in the texts and the percentage of plagiarism. Texts consisting of a single paragraph demonstrated the lowest plagiarism rate. This suggests that presenting the source texts within a single coherent unit allows ChatGPT to better understand and paraphrase the content effectively. Dividing the text into separate paragraphs may lead to fragmented understanding and potentially contribute to higher levels of plagiarism.

It is worth noting that the prompts used in our study did not yield significant differences in the levels of plagiarism. This indicates that the specific prompt provided to ChatGPT does not significantly influence its paraphrasing capability. In addition, this outcome might be the consequence of the bot’s strong ability to understand our true intentions when issuing commands, or it might be because our command words were brief or similar to one another. However, further investigation into the effect of different prompts and their impact on plagiarism is warranted to explore this aspect in more detail.

ChatGPT has a wide range of applications that can be effectively used. Numerous articles have discussed the use of ChatGPT in composing scientific literature, with a particular study illustrating its capability to generate formal research articles. The researchers observed that the language used is articulate, adopts a conventional tone, and offers a pleasant reading experience [19].

ChatGPT has the potential to serve as a search engine that directly responds to queries, eliminating the need to navigate to external sites for information. This streamlines the process of writing research papers, reducing the time spent by authors on the often arduous task of searching for articles and applying various selection criteria. This, in turn, allows authors to dedicate more time to their actual research work and methodology [20].

Moreover, articles created by ChatGPT seem to elude traditional plagiarism detection methods. In a research study, the chatbot was tasked with generating 50 medical research abstracts using a subset of articles. The resulting articles underwent examination by plagiarism detection software, an AI-output detector, and a panel of medical researchers who were tasked with identifying any artificially generated abstracts. The findings revealed that abstracts generated by ChatGPT seamlessly passed through the plagiarism detection software, registering a median originality score of 100%, indicating the absence of detected plagiarism. In contrast, the AI-output checker only identified 66% of the generated abstracts [21].

While ChatGPT and other AI tools hold promise in various applications, their deployment in medical writing raises ethical and legal considerations. These concerns encompass potential violations of copyright laws, medico-legal complexities, and the risk of inaccuracies or biases in the generated content. It is crucial to recognize and confront the limitations and challenges linked to the use of AI in medical writing [20,22,23].

### Limitations and Future Suggestions

The sample size used in our study was relatively small, and as a result, we recommend that future investigations incorporate larger sample sizes to enhance the robustness of the findings. It is worth noting that our study was conducted using ChatGPT version 3.5, which was a publicly available version at the time of our research. Unfortunately, we did not have access to ChatGPT version 4, preventing us from evaluating the efficacy of this updated version in terms of paraphrasing capabilities.

It is essential to acknowledge that our study exclusively focused on providing medical content to ChatGPT. We encourage other researchers to explore the impact of using different content types on the efficacy of ChatGPT. This would allow for a comprehensive understanding of whether the effectiveness of ChatGPT is influenced by the specific domain or topic of the content it receives. Conducting such investigations will provide valuable insights into the generalizability and adaptability of ChatGPT across various subject matters.

Moreover, a recognized limitation of ChatGPT is its tendency to produce inconsistent results with the same prompts [24]. To relatively address this challenge, we used a comprehensive approach. Each prompt was provided with nine texts, varying paragraph structures (text one with 1 paragraph, text one with 3 paragraphs, text two with 1 paragraph, text two with 3 paragraphs, text two with 5 paragraphs, text three with 1 paragraph, text three with 3 paragraphs, text three with 5 paragraphs, and text three with 7 paragraphs). Furthermore, we requested ChatGPT to paraphrase each of these texts twice using the same prompt. We then calculated the mean plagiarism rates for both the first and second attempts, along with the overall mean plagiarism rate for each prompt (Table 2).

Nevertheless, we recommend that future studies take this limitation into account and explore additional measures to enhance the robustness of assessments. Specifically, researchers may consider providing ChatGPT with a greater number of texts exhibiting different paragraph structures and incorporating a higher frequency of repetitions in the paraphrasing process.

We used similar prompts and provided them to ChatGPT. We recommend that future studies adopt a broader range of prompts to assess ChatGPT’s performance across different input variations. This approach allows for a more comprehensive evaluation and facilitates the identification of optimal prompts to minimize plagiarism rates.

An important consideration with ChatGPT lies in the potential for hallucination and biases, particularly in the generation of medical content [25]. In our study, two independent researchers evaluated the content provided by ChatGPT, comparing it with the original texts. However, we acknowledge that the texts used in our assessment may not have been sufficiently complex. To address this limitation, we recommend that future studies incorporate both simple and more intricate texts to thoroughly evaluate the biases that ChatGPT may introduce during the paraphrasing of medical content. This approach will provide a more nuanced understanding of the model’s performance.

### Conclusion

While ChatGPT has been shown to significantly reduce plagiarism in texts, it is important to note that the resulting plagiarism rates of the provided texts may still be considered high, which may not meet the acceptance criteria of most scientific journals. Therefore, medical writers and professionals should carefully consider this issue when using ChatGPT for paraphrasing their texts. There are a couple of strategies authors can use to improve the paraphrasing efficacy of ChatGPT. Presenting the texts in a single-paragraph format and repeating the requesting procedure with ChatGPT. By considering these strategies and being mindful of the potential limitations, authors can strive to improve the paraphrasing efficacy of ChatGPT and address the challenge of high plagiarism rates associated with its outputs.

## Abbreviations

AI: artificial intelligence
